# Brachial Plexus Wrapping by Free Perforator Fat Flap for Treatment of Recurrent Neurogenic Thoracic Outlet Syndrome: A Case Report

**DOI:** 10.1002/ccr3.72945

**Published:** 2026-06-23

**Authors:** F. Thuau, G. Gadbled, B. Pradier, P. Perrot, U. Lancien

**Affiliations:** ^1^ Plastic Reconstructive, and Aesthetic Surgery Department, CHU Nantes Nantes University Nantes France; ^2^ INSERM UMRS 1229, Laboratory Regenerative Medicine and Skeleton (RMeS) Nantes France; ^3^ Orthopedic and Traumatology Surgery Department, CHU Nantes Nantes University Nantes France; ^4^ Department of Radiology, CHU Nantes Nantes University Nantes France

**Keywords:** brachial plexus, case report, free flap, nerve wrapping, neurogenic thoracic outlet syndrome

## Abstract

A 35‐year‐old woman with recurrent neurogenic thoracic outlet syndrome underwent supraclavicular neurolysis and brachial plexus wrapping using a free DIEP flap. At 2‐year follow‐up, pain and functional scores (VAS, DASH, SF‐36) significantly improved. Free fat flaps offer a low morbidity option for reducing perineural fibrosis in RNTOS.

## 1 Introduction

Neurogenic thoracic outlet syndrome (NTOS) accounts for approximately 90% of all forms of thoracic outlet syndrome [1]. Although the standard surgical treatment combines anterior scalenectomy with first rib resection, symptoms recur in 5% to 30% of operated patients [2, 3]. These recurrences are frequently driven by excessive perineural fibrosis, causing nerve adhesion and compression of the brachial plexus. Reoperations typically combine brachial plexus neurolysis with resection of residual anatomical conflicts [4]; however, nerve release alone carries a high risk of recurrent fibrosis. Flap coverage is therefore classically recommended to enhance local vascularization, protect nerves from external compression, and limit scar reformation [5]. A recent systematic review published by our team identified eleven distinct brachial plexus wrapping techniques, highlighting the diversity of available approaches but the absence of consensus on the optimal strategy [6]. The classic option—the pedicled latissimus dorsi muscle flap—requires extensive regional dissection, adding morbidity to an already compromised surgical field. In contrast, adipose perforator flaps spare the muscle and minimize donor site sequelae, while free transfer avoids additional scarring in the cervical region [7].

We report the first case of brachial plexus wrapping using a free Deep Inferior Epigastric Perforator (DIEP) flap for recurrent NTOS (RNTOS), supported by our systematic review which identified no previously published similar case.

## 2 Case History/Examination

### 2.1 Initial Presentation and Conservative Management

A 35‐year‐old right‐handed female patient, employed as an educational assistant, presented with non‐traumatic bilateral NTOS, with symptoms predominantly on the left side. At the time of consultation, she had been on medical leave for 2 years. Clinical examination revealed diffuse left periscapular pain radiating to the entire upper limb and a bilaterally positive Roos test. Electromyography (EMG) showed C8‐T1 nerve root involvement on the left. Dynamic vascular imaging (Doppler ultrasound and MRI) confirmed arterial, venous, and neural compression within the costoclavicular space and the interscalene triangle.

Initial management consisted of several months of intensive inpatient rehabilitation. This multidisciplinary program included daily physical therapy focused on postural correction, periscapular muscle strengthening, stretching to prevent retractions, respiratory retraining, and maintenance of joint mobility. Despite these efforts, symptoms persisted, leading to the decision for surgical intervention.

### 2.2 Primary Surgery and Early Postoperative Course

Two years prior to her referral to our department, the patient underwent surgery at another institution. The procedure included an anterior scalenectomy, first rib resection, and brachial plexus neurolysis via a supraclavicular approach. While the immediate postoperative course was uneventful, the symptomatic relief was short‐lived.

### 2.3 Recurrence and Secondary Complications

Within months of the initial surgery, the patient developed new neurological deficits, including hypoesthesia in the supraclavicular nerve territory and left scapulothoracic dyskinesis. She reported persistent neuropathic pain (C8‐T1 distribution) and developed signs of ulnar nerve palsy (ulnar claw deformity, intrinsic muscle weakness, and impaired flexion of the 4th and 5th digits).

Follow‐up EMG confirmed worsening C8‐T1 dysfunction with no voluntary motor activity in the abductor digiti minimi. Dynamic vascular imaging demonstrated complete resolution of the previous vascular compression, thereby excluding vascular recurrence and confirming an RNTOS.

### 2.4 Intermediate Management and Failed Revisions

A second intensive three‐month rehabilitation program was initiated, alongside a specialized pain management protocol. This included Capsaicin patches applied every 3 months to the supraclavicular area; intravenous ketamine infusions and corticosteroid injections targeting the periscapular muscles.

Nine months after the primary surgery, a left pectoralis minor tenotomy was performed via a deltopectoral approach to address persistent scapular tilting. However, no significant symptomatic improvement was achieved.

### 2.5 Diagnostic Workup for Revision Surgery

**FIGURE 1:**
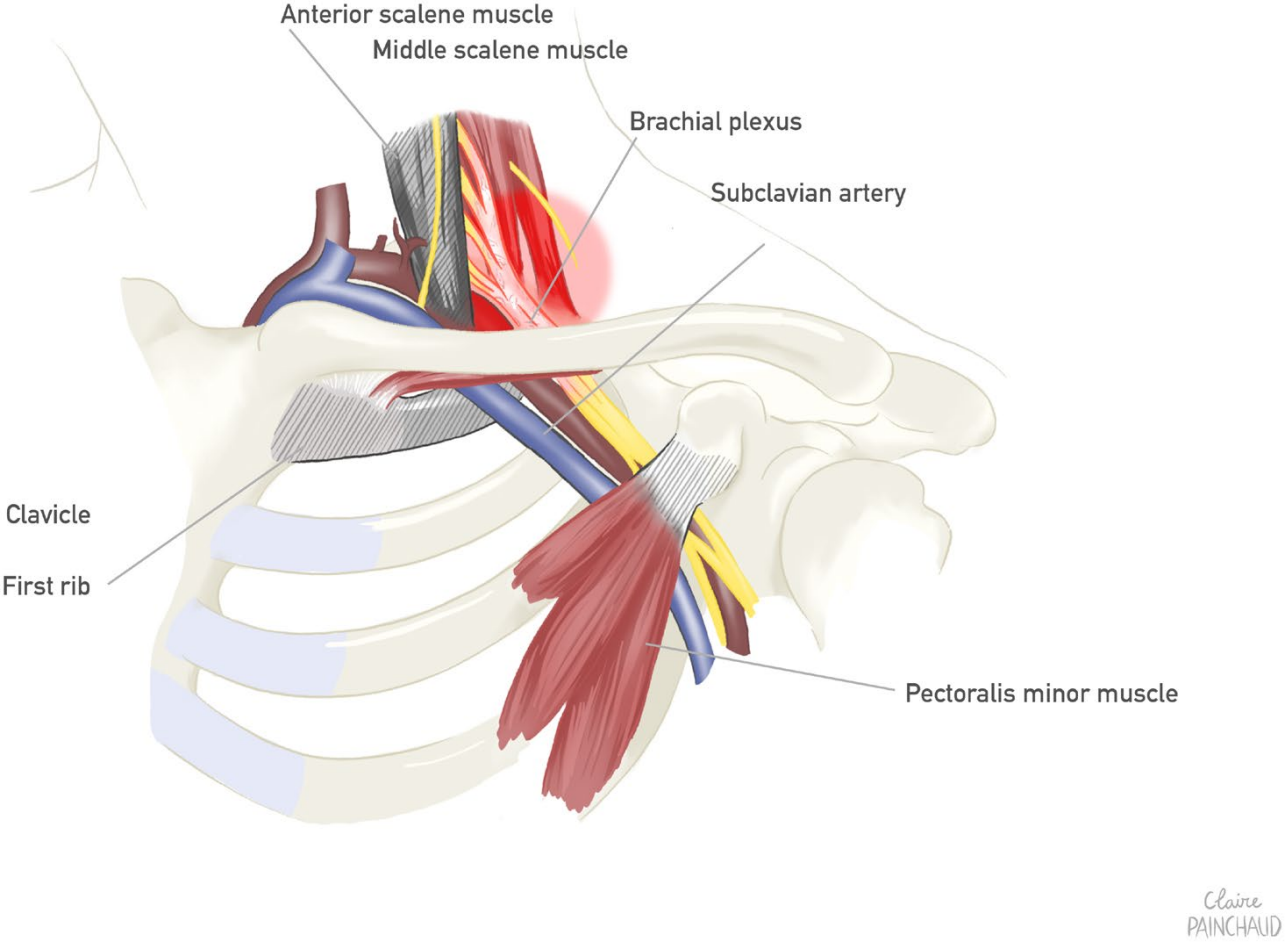
Schematic representation of the patient's preoperative condition. In gray: The anterior scalene muscle, first rib, and pectoralis tendon, which were resected during previous surgeries. In red: Brachial plexus impairment due to perineural scar fibrosis.

Given the failure of conservative and intermediate surgical treatments, a multidisciplinary revision was planned. Post‐surgical Parsonage‐Turner syndrome was ruled out due to the chronic, non‐remitting nature of the symptoms over 2 years. MRI provided evidence of significant perineural scar tissue tethering the brachial plexus (Figure 1, Figure S1).

### 2.6 Surgical Procedure: Multidisciplinary Approach

**FIGURE 2:**
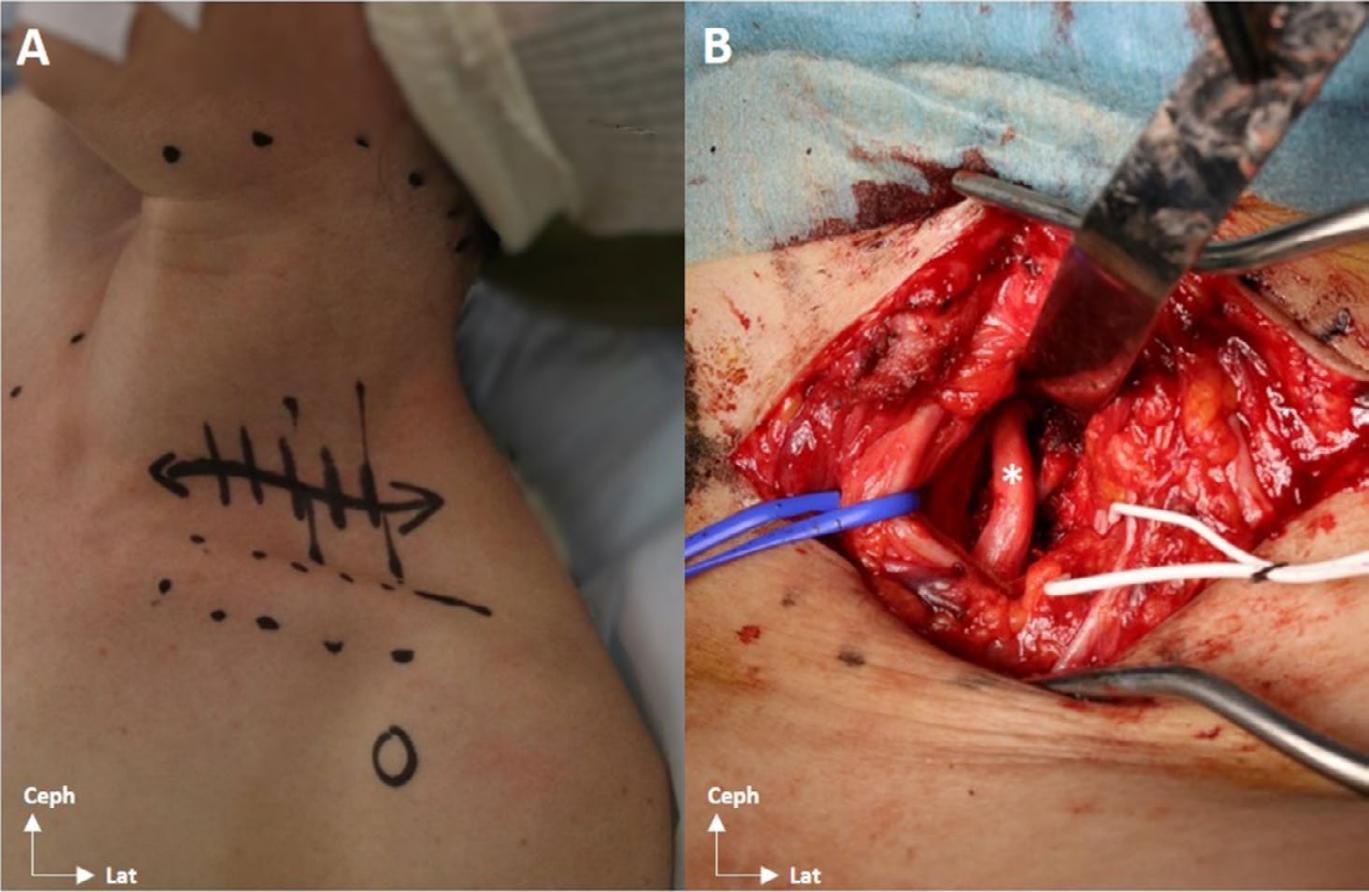
(A) Supraclavicular approach. (B) Intraoperative view following extensive neurolysis. The star (*) indicates the lower trunk.

The definitive revision was performed simultaneously by two surgical teams. The orthopedic team first addressed the scapulothoracic dyskinesis by performing a scapulopexy using a gracilis tendon graft reinforced with FiberTape (Arthrex, Naples, FL, USA). Access was gained through the pre‐existing supraclavicular scar. Intraoperative findings revealed severe fibrosis encasing the middle (C7) and lower (C8‐T1) trunks. This scar tissue carried a high risk of nerve injury. However, meticulous neurolysis of the middle and lower trunks as well as the long thoracic nerve was performed (Figure 2), while the phrenic nerve was carefully identified and protected.

Simultaneously, the plastic surgery team harvested a free DIEP flap from the abdominal region. A DIEP was selected due to the necessity of obtaining a long vascular pedicle, unlike a Superficial Circumflex Iliac Perforator (SCIP) flap [8], and to leave a more discreet scar for this young female patient. Indeed, this abdominal scar remains well‐concealed beneath undergarments, unlike an Anterolateral thigh (ALT) flap scar [9]. Furthermore, preoperative CT angiography had confirmed favorable DIEP perforators, demonstrating a short intramuscular course and an optimal caliber. During harvest, the rectus abdominis muscle was completely spared, and a 10‐cm vascular pedicle was isolated. A small skin paddle was preserved on the flap for postoperative clinical monitoring.

**FIGURE 3:**
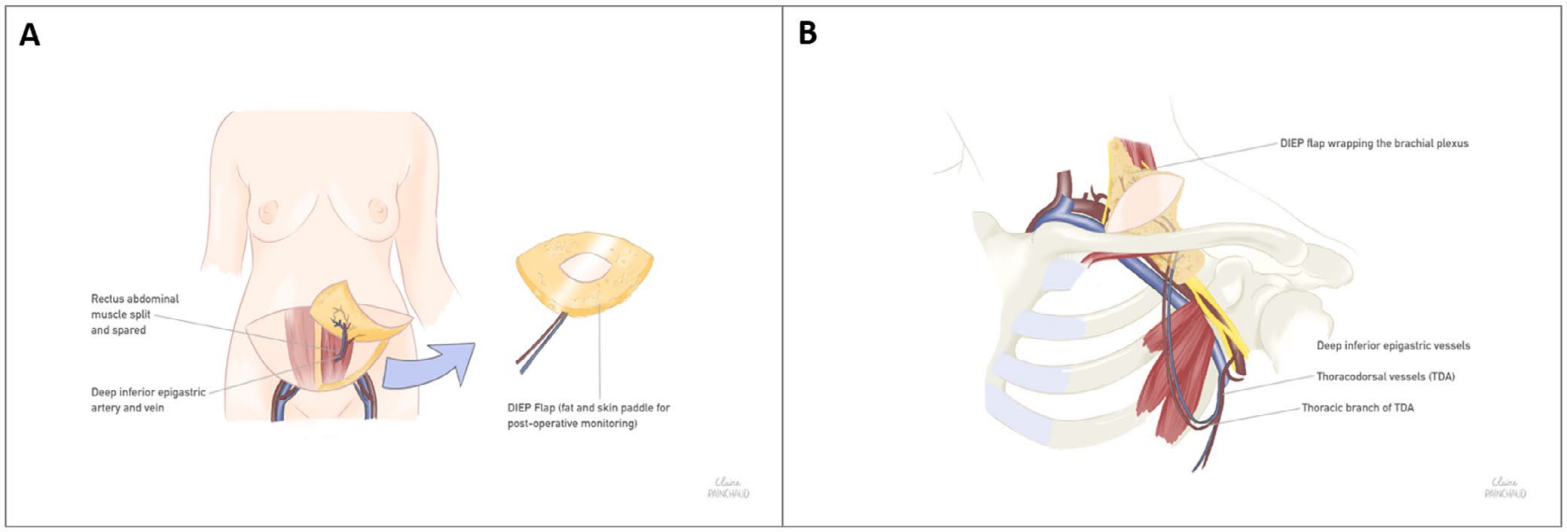
(A) Abdominal harvesting of a free DIEP flap. A skin paddle is preserved for postoperative monitoring. (B) Schematic representation of brachial plexus neurolysis and wrapping with the adipose flap. Vascular anastomoses are performed at the axillary level using the thoracodorsal vessel's thoracic branches.

To avoid inducing secondary mechanical compression on the released nerves, precise measures were taken during the inset. The harvested DIEP flap was meticulously defatted intraoperatively, preserving only a pliable fat layer centered on the perforator, which reduced its total volume to approximately 12 × 6 cm with a 2.5 cm thickness. Instead of a tight circumferential constriction, the flap was positioned to wrap the brachial plexus roots as a loose hammock, covering the C7, C8, and T1 trunks in a 270‐degree wrapping (Figure 3). It was securely anchored deeply onto the fascia of the remnant scalene muscles using interrupted 4–0 Vicryl sutures, ensuring no tension or secondary constriction.

Due to prior surgery, no suitable or safe recipient vessels were available locally in the neck. This primary intraoperative challenge necessitated an extension of the operative field via an axillary approach to perform a tension‐free microanastomosis onto the thoracic branch of the thoracodorsal vessels. The 10‐cm pedicle was safely tunneled beneath the pectoralis major muscle to reach the axillary recess. The overall flap ischemic time was 86 min. An ON‐Q PainBuster catheter and a local drain were placed before closure.

### 2.7 Postoperative Course and Follow‐Up

The patient was monitored in the intensive care unit (ICU) for 24 h. Rehabilitation began on postoperative day four with isometric strengthening of the periscapular muscles; however, resisted movements of the left upper limb were restricted for 6 weeks to prevent scapulopexy failure. The patient wore an elbow‐to‐body orthosis during this initial 6‐week period, and no immediate postoperative complications were noted. Early specialized rehabilitation was subsequently initiated.

**FIGURE 4:**
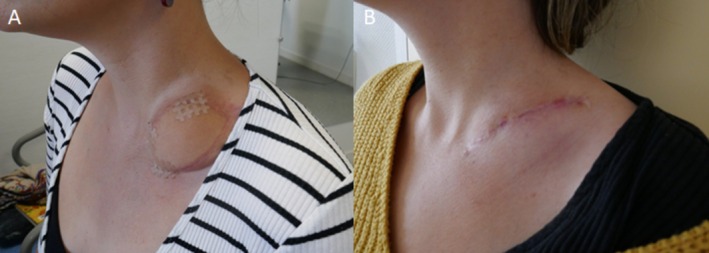
(A) Postoperative view showing the flap skin paddle. (B) Debulking and excision of the skin paddle performed 9 months after the initial procedure.

A secondary debulking procedure was intentionally planned and performed at 9 months to safely excise the monitoring skin paddle and further thin the subcutaneous tissue once revascularization and local tissue healing had fully stabilized (Figure 4).

The complete timeline of clinical events, interventions, and outcomes for this case is summarized in Table 1.

**TABLE 1:** Timeline.

Time period	Clinical status & interventions	Key outcomes
Year −2	Diagnosis of bilateral NTOS; intensive inpatient rehabilitation (6 months)	Failure of conservative management
T0 (Primary Surgery)	Initial decompression: Supraclavicular scalenectomy + first rib resection + neurolysis	Uneventful immediate recovery
Months 3–24	Symptom recurrence Intensive physiotherapy in rehabilitation center and chronic pain management	EMG: Worsening C8‐T1 dysfunction MRI confirms perineural fibrosis
Month 9	Secondary surgery: Left pectoralis minor tenotomy	No significant symptomatic improvement
Year 2 (Revision)	Multidisciplinary Surgery: Scapulopexy + Brachial plexus neurolysis + Free DIEP flap wrapping	Successful microsurgical transfer
Year 2 + 9 months	Scheduled debulking of the flap and skin paddle excision	Improved shoulder contour and volume
Year 4 (Final Follow‐up)	Final 2‐year post‐revision assessment.	Significant improvement: VAS: 70 to 45; QDSA: 32 to 14 DASH: 68.3 to 49.2 Improved QoL (SF‐36) and HADS scores Reduced analgesic intake

## 3 Conclusion and Results

Annual follow‐up demonstrated stable outcomes at 2 years, with no RNTOS. Validated patient‐reported outcome measures were administered preoperatively and at 2 years postoperatively to assess pain, health‐related quality of life, upper‐limb function, and symptoms of anxiety and depression.

Maximum pain intensity on a 0–100 mm visual analog scale (VAS) over the preceding 8 days decreased from 70 mm preoperatively to 45 mm at follow‐up. Qualitative pain assessment was conducted using the abridged version of the St‐Antoine Pain Questionnaire (QDSA) [10], an adaptation of the short‐form McGill Pain Questionnaire, which evaluates 16 pain descriptors on a scale from 0 (no pain) to 4 (extremely severe). Thirteen of 16 descriptors improved between the preoperative and two‐year evaluation, and the total QDSA score decreased from 32 to 14.

Quality of life (QoL) assessment using the SF‐36 questionnaire showed improvements at 2 years postoperatively in seven of eight domains: physical functioning (PF: 40 to 55), body pain (BP: 20 to 45), general health (GH: 70 to 90), social functioning (SF: 12.5 to 50), mental health (MH: 44 to 80), vitality (25 to 45), role limitations due to emotional problems (RE: 0 to 100); while role limitations due to physical health (RP) remained unchanged at 0.

The DASH score improved from 68.3 preoperatively to 49.2 at 2 years [11]. On the Hospital Anxiety and Depression Scale (HADS), depression scores decreased from 15 to 6 and anxiety from 10 to 7, both within the normal range [12].

At 2 years postoperatively, the patient had significantly reduced her analgesic consumption, although it remained daily. She no longer required tramadol, nefopam, gabapentin, amitriptyline, ketamine, or topical lidocaine and capsaicin patches. However, she continued to use paracetamol (alone or combined with opium powder) and non‐steroidal anti‐inflammatory drugs, and she employed transcutaneous electrical nerve stimulation (TENS).

## 4 Discussion

Our study is the first to report a case of brachial plexus nerve wrapping using a free perforator fat flap for the treatment of RNTOS. We provide a detailed description of the surgical procedure and present a comprehensive prospective multimodal pain assessment, in accordance with the recommendations for evaluating neuropathic pain in the upper limb described by Novak & Katz [13].

### 4.1 Outcome Interpretation

All assessed parameters showed improvement at 2 years postoperatively. The DASH score improved by 19.1 points (68.3 to 49.2), exceeding the minimal clinically important difference (MCID) of 12 to 14 points established for this instrument indicating a meaningful gain in upper limb function [14]. Similarly, improvements in SF‐36 domains exceeded the commonly reported MCID of approximately 5 per domain in seven of the eight improved domains [15]. HADS scores for both depression (−9) and anxiety (−3) surpassed the accepted MCID of 1.5 to 2 points, returning to the normal range [15].

The VAS improvement of 25 mm (70 to 45) exceeds the established MCID of approximately 10 mm [16] and therefore represents a clinically meaningful change. However, the absolute residual pain level (45/100) remains significant and warrants discussion. In this patient, pain had a predominantly neuropathic component, as evidenced by the high preoperative QDSA score (32/64) and the pattern of C8–T1 involvement on electromyography. This pathophysiological distinction explains the apparent dissociation between a modest VAS improvement and the more substantial gains observed in neuropathic pain descriptors assessed by the QDSA (13 of 16 descriptors improved; total score 32 to 14), analgesic consumption, and QoL. The VAS, as a unidimensional scale capturing global pain intensity, is less sensitive than the QDSA to qualitative changes in neuropathic symptomatology, and should therefore not be interpreted in isolation in this context.

This was a complex case involving a multiply operated patient with chronic pain and high preoperative analgesic consumption. Persistent pain phenomena were therefore expected after surgery, and daily analgesic use persisted due to altered and complex neurological pain patterns [17, 18, 19]. Despite these limitations, the overall trajectory across all domains remains encouraging, although no definitive conclusions can be drawn from a single experience given the multiple confounding factors.

### 4.2 Comparison With Existing Techniques

Brachial plexus wrapping techniques can be organized into three categories: synthetic materials, non‐vascularized biological tissues, and vascularized flaps (Table 2) [6].

**TABLE 2:** Summary of brachial plexus wrapping techniques.

Category	Technique	Main indication	Key advantage	Main limitation
Synthetic materials	HA membrane [20]	RNTOS	Simple, no donor site	No biological effect, no vascularisation
	PLA film [3]	RNTOS	Simple, no donor site	No biological effect, no vascularisation
Non‐vascularized biological	Amniotic membrane [21]	RNTOS	Anti‐fibrotic, anti‐inflammatory	No vascularization, limited data
	Collagen matrix/Vein [22, 23]	Peripheral nerves	Low morbidity	Not reported for brachial plexus
Local vascularized flaps	Scalene fat pad [24]	RNTOS	Same operative field	Often fibrotic in revision, uncertain viability after TCA ligation
	Adipofascial deltopectoral [25]	RNTOS/RIBP	No muscle sacrifice, no microsurgery	Limited arc of rotation, local dissection
	Retropectoral adipofascial [26]	RNTOS/RIBP	Large surface area	Requires pectoralis major detachment
	Latissimus dorsi [5]	RNTOS/RIBP	Reliable, well described	Muscle sacrifice, extensive dissection, donor site morbidity
	Serratus anterior [27]	RNTOS	Proximity	Risk of scapular winging
Free vascularized flaps	Greater omentum [28]	RIBP	Excellent anti‐fibrotic properties, large surface	Laparotomy, hernia risk, significant morbidity
	Groin flap [29]	RIBP	Low morbidity, hidden scar	Short/variable pedicle
	Free DIEP flap (present case)	RNTOS	Long pedicle, muscle‐sparing, hidden scar, no regional dissection	Microsurgical expertise required, longer operative time, ICU monitoring

Abbreviations: HA, Hyaluronic Acid; PLA, Polylactic Acid; RIBP, Radiation‐Induced Brachial Plexopathy; RNTOS, Recurrent Neurogenic Thoracic Outlet Syndrome; TCA, Transverse Cervical Artery.

Synthetic and non‐vascularized biological materials act as a physical barrier to reduce perineural adhesions but provide no vascularization. Hyaluronic acid membranes, PLA films, and amniotic membrane have all been reported in NTOS and RNTOS with variable results [3, 20, 21]. While amniotic membrane shows promising anti‐fibrotic properties, none of these options address the local tissue hypoxia induced by repeated surgery, which drives fibrotic recurrence.

Local vascularized flaps provide this vascularization but introduce specific constraints in the revision setting. All require extensive dissection in an already scarred field, risking renewed nerve adhesions. Additionally, muscle‐based options such as the latissimus dorsi add donor site morbidity to an upper limb already functionally compromised [5, 30], while the scalene fat pad is frequently fibrotic and of uncertain viability after transverse cervical artery ligation [24].

Free vascularized flaps bring well‐vascularized tissue from an unaffected donor site, avoiding additional cervical dissection. The greater omentum has demonstrated excellent results in radiation‐induced plexopathy but requires laparotomy with significant abdominal morbidity [28, 31]. Among free perforator flaps, the DIEP was selected for its long and reliable pedicle enabling tension‐free axillary anastomosis in a scarred field, while sparing the rectus abdominis muscle and concealing the donor site scar within undergarments. An ALT flap could provide comparable pedicle length; however, the DIEP offers a superior aesthetic outcome. The SCIP flap, while thinner and potentially better suited for nerve wrapping, has a typically short and anatomically variable pedicle that may preclude tension‐free anastomosis to distant recipient vessels [8]. This approach does, however, necessitate extended operative time, a double surgical team with microsurgical expertise, postoperative ICU monitoring, and careful assessment of recipient vessel availability—particularly when the thyrocervical trunk or transverse cervical artery has been previously resected.

### 4.3 Limitations

Several limitations of this report must be acknowledged. First, the single‐case design precludes any definitive conclusions regarding the efficacy of this technique, and the absence of a comparator prevents attribution of the observed improvements to the surgical intervention alone. Second, this case involved a highly selected patient treated in a tertiary referral center with dedicated microsurgical and orthopedic expertise, limiting the generalizability of the findings. Third, the complexity of the clinical presentation, including chronic pain, central sensitization, and high preoperative analgesic consumption, introduces multiple confounding factors that make outcome interpretation particularly challenging.

Regarding patient selection, this technique should be considered in multiply operated RNTOS patients with MRI‐confirmed perineural fibrosis, failure of conservative management, and absence of suitable local flap options or recipient vessels in the cervical region. The availability of an adequate abdominal donor site is also required for DIEP flap harvest. Future case series with standardized outcome measures and longer follow‐up are needed to define the indications more precisely and assess the reproducibility of this approach.

## Author Contributions

**B. Pradier:** formal analysis, software. **G. Gadbled:** data curation, validation. **P. Perrot:** writing – review and editing, validation. **F. Thuau:** data curation, writing – original draft, writing – review and editing, methodology, conceptualization. **U. Lancien:** data curation, supervision, investigation, visualization, project administration, resources, funding acquisition.

## Funding

The authors have nothing to report.

## Ethics Statement

As a single‐case report with the patient's signed consent, no additional ethical review was required.

## Consent

The patient provided written informed consent for the use of her clinical data and images for publication and presentation.

## Conflicts of Interest

The authors declare no conflicts of interest.

## Supporting information


**Figure S1:** (A) Coronal STIR T2‐weighted MRI; P, left brachial plexus stretched by scar tissue. (B) Coronal T1‐weighted MRI; S, fibrotic scar adherence.

## Data Availability

Data sharing not applicable to this article as no datasets were generated or analyzed during the current study.
