# Supplementary figures and images for: Brachial Plexus Wrapping by Free Perforator Fat Flap for Treatment of Recurrent Neurogenic Thoracic Outlet Syndrome: A Case Report
Source: Clin Case Rep. 2026 Jun 23;14(7):e72945. doi: 10.1002/ccr3.72945 (PMC13290698; doi:10.1002/ccr3.72945)

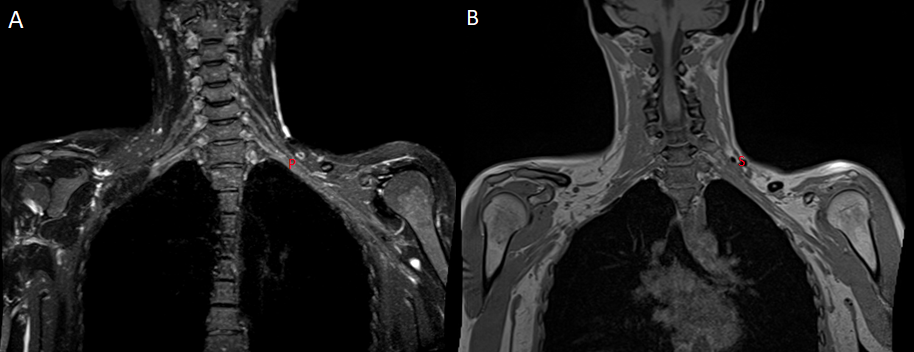

Supplement: Supplementary file 1 — Figure S1: (A) Coronal STIR T2‐weighted MRI; P, left brachial plexus stretched by scar tissue. (B) Coronal T1‐weighted MRI; S, fibrotic scar adherence. [file CCR3-14-e72945-s001.tif]
